# More complicated than it seems: The diversity of cryptococcal glucuronoxylomannan

**DOI:** 10.1371/journal.ppat.1011521

**Published:** 2023-08-03

**Authors:** Bianca A. G. Sena, Luisa J. Jozefowicz, Marcio L. Rodrigues

**Affiliations:** 1 Instituto Carlos Chagas, Fundação Oswaldo Cruz (Fiocruz), Curitiba, Brazil; 2 Instituto de Microbiologia Paulo de Góes (IMPG), Universidade Federal do Rio de Janeiro, Rio de Janeiro, Brazil; 3 Programa de Pós-Graduação em Biologia Parasitária, Instituto Oswaldo Cruz, Fiocruz, Rio de Janeiro, Brazil; Vallabhbhai Patel Chest Institute, INDIA

## 1. Structure and functions of a eukaryotic capsule

Polysaccharides are complex carbohydrates composed of more than a dozen monosaccharide units connected by glycosidic linkages [1]. Unlike proteins and nucleic acids, the cellular synthesis of polysaccharides does not involve the typical scaffolds used in transcriptional and translational processes [1]. This particularity implies that there are no codon-like structures to regulate the termination of polysaccharide synthesis. Consequently, a general characteristic of microbial polysaccharides is their heterogeneity in molecular mass, resulting in molecules with identical composition but variable dimensions.

Microbial capsules made of polysaccharides are surface structures often associated with prokaryotes. *Cryptococcus* is the eukaryotic genus that most commonly presents this characteristic [2]. The capsule is essential for members of the *Cryptococcus* genus to survive in the environment, offering protection against free radicals and dehydration [2]. In addition, the cryptococcal capsule is a key element in the interaction of pathogenic cryptococci with the immune system [2]. Although the capsule is not essential for the regular growth of *Cryptococcus*, it is required for virulence [3].

The cryptococcal capsule is mainly formed by 2 complex polysaccharides. Glucuronoxylomannan (GXM), the most abundant component, accounts for approximately 90% to 95% of the capsular composition [4]. Glucuronoxylomannogalactan (GXMGal), the second most abundant capsular polysaccharide, corresponds to 5% to 8% of the capsule composition [4]. These polysaccharides are synthesized through the polymerization of sugar monomers obtained from their nucleoside forms, namely uridine diphosphate (UDP)-glucuronic acid, UDP-xylose, UDP-galactose, and guanosine diphosphate (GDP)-mannose, by a collection of glycosyltransferases predominantly located in the Golgi apparatus [4]. In contrast to prokaryotic polysaccharides, which are usually polymerized at the cell surface level, there is evidence that GXM polymerization starts in the cytoplasm for further export to the cell surface level in post-Golgi secretory vesicles [5]. GXM is then assembled into the capsule by mechanisms that have been extensively discussed before [4]. The surface location and polymerization sites of GXMGal are still not fully known. Mannoproteins are also part of the capsule composition. However, their distribution within the capsule is still uncertain, and they account for a minor part of the capsule constitution [2].

## 2. Structural and functional diversity of cryptococcal GXM

The structural arrangement of GXM components (glucuronic acid, mannose, and xylose) has led to the characterization of 4 capsular serotypes (A, B, C, and D), which have been used for decades in the classification of pathogenic cryptococcal isolates [4]. It is now clear that serotype classification obscures intriguing structural properties of the capsule. For instance, studies with 3 isolates known to belong to the C serotype showed variable patterns of reactivity with monoclonal antibodies raised to GXM [6]. All 3 isolates produced a capsule, but immunofluorescence tests revealed that one of the isolates did not react with any of the antibodies tested, suggesting unique serological properties. Two of the isolates were recognized by the same antibody, but the microscopic patterns of antibody binding were diverse. While an annular pattern of antibody binding was observed in one of the isolates, a punctuate pattern of serological reactivity was observed for the second isolate [6]. This diversity implies that, although supposedly similar in serotype, GXM in these strains manifested serological and, consequently, structural differences.

**Fig 1:**
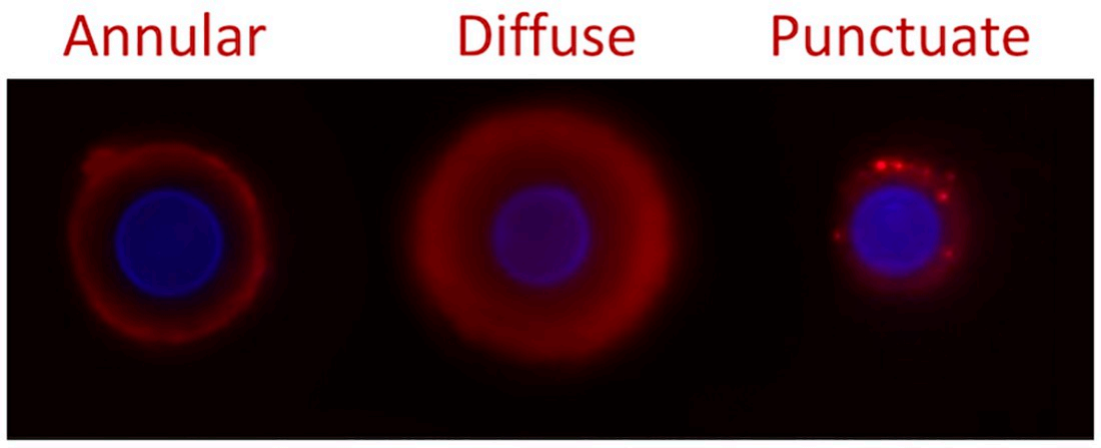
Patterns of staining of the cryptococcal capsule with antibodies raised to GXM. Our literature search indicated that 3 patterns predominate, namely annular, diffuse, and punctuate. Images were obtained in our laboratory. Red fluorescence indicates capsule staining, while the blue fluorescence indicates the cell wall. For protocol details, please see [23].

Two subsequent studies using a larger collection confirmed the lack of relationship between serotype classification and reactivity with antibodies to GXM [7,8]. Specifically, immunofluorescence and flow cytometry analyses of 24 *C*. *neoformans* isolates (VNI genotype, serotype A) showed highly variable profiles of antibody binding to the capsule [7], reaffirming the notion that serotype classification misses structural diversity. Capsule sizes and the concentration of extracellular GXM were also variable, confirming the high diversity of capsular structures [7]. This diversity was confirmed by a follow-up study that included 32 strains of the *C*. *neoformans* and *C*. *gattii* complexes, as concluded by immunofluorescence using anti-GXM antibodies, analysis of capsule size, and determination of extracellular GXM [8]. The patterns of GXM staining observed in these studies and others are illustrated in Fig 1. Together, these data reinforce the notion that, although helpful for decades, the serotype-based classification of *Cryptococcus* misses important structural and functional features of the cryptococcal capsule.

Another important factor that influences the determination of the biological properties of GXM is the method used for its extraction. For example, the extraction of capsular GXM using gamma radiation or dimethyl sulfoxide resulted in significant differences in glycosyl composition, molecular mass, polysaccharide dimensions, charge, viscosity, circular-dichroism spectra, and reactivity with monoclonal antibodies [9]. Additionally, the analysis of extracellular, soluble GXM showed that polysaccharide precipitation with cationic detergent resulted in larger molecular masses and a different conformation compared to polysaccharide fractions obtained by filtration [9]. Alterations in the molecular mass of GXM were correlated with important functional changes. For instance, coating experiments in which acapsular cells were used to incorporate GXM into the cell surface demonstrated that the coating pattern depends on the molecular mass [10]. GXM fractions ranging from 1 to 100 kDa tended to accumulate at the cell division area, while fractions of larger masses (100 to 300 and >300 kDa) produced a dotted, punctuate pattern of GXM incorporation into the entire cell surface. The typical annular, uniform pattern of GXM coating after incorporation by acapsular cells was only achieved when molecules of 1 to higher than 300 kDa were combined, suggesting that the assembly of GXM into the cell surface requires a combination of polysaccharides with a wide range of molecular mass [10].

The variations in molecular mass apparently have no effect on binding to host cell receptors but influence cytokine production by host cells. Fluorescence-based assays using monoclonal antibodies to GXM revealed that polysaccharide fractions of variable masses (10 to 100; 100 to 300; >300 kDa, and all of them in combination) produced similar levels of association with macrophages [10]. Similar tests using macrophages lacking the well-characterized GXM receptors TLR2 and CD14 showed that both the percentage of GXM-containing macrophages and the indices of GXM-derived fluorescence intensity were similar [10], suggesting that the molecular mass of GXM does not influence its interaction with host receptors. As for the macrophage responses in vitro, quantification of TNF-α, RANTES, IL-10, and IL-6 revealed that for all cytokines tested, the fraction with a molecular mass >300 kDa manifested the lowest effectiveness in stimulating cellular responses compared to the other polysaccharide samples, while fractions containing the full molecular mass range of GXM were the most efficient stimulators of all cytokines tested [10]. The diversity of cytokine production depending on the molecular mass of GXM was also observed in the lungs of mice stimulated with different polysaccharide fractions. In this model, the most pronounced differences were observed when IL-10 was assessed. While the full molecular mass range sample caused a reduction in the basal levels of lung IL-10, the 10- to 100-kDa fraction had no effect on the levels of this cytokine compared to PBS-treated animals. The >300 kDa sample, however, induced markedly increased levels of IL-10 production in the lung compared to animals stimulated with 10 to 100 kDa fractions or the full molecular mass sample, confirming the notion that molecular mass is an important factor in determining the functions of GXM [10]. These results support previous observations showing that GXM samples of different molecular masses produced by *C*. *gattii* and *C*. *neoformans* vary in their ability to induce nitric oxide production [11].

Little is known about how these differences impact the course of cryptococcosis. However, due to the lack of molecular markers necessary to halt the molecular polymerization of polysaccharides, it is expected that the significant variability in the molecular mass of GXM observed in vitro will also occur in vivo. This supposition is supported by the observation that the dimensions of the capsule change in vivo depending on the infected organ [12]. The impact of this diversity during infection remains to be determined.

## 3. GXM-like molecules in other fungal pathogens

GXM is the molecular product of the sequential activity of Golgi-associated glycosyltransferases [4]. These enzymes are abundant and fully functional in several eukaryotes, including fungi. Therefore, it is not unlikely that other fungal species can synthesize polysaccharides that resemble GXM in structure.

An early report on the production of a monoclonal antibody to cryptococcal GXM revealed that, among several yeasts tested, cross-reaction with the antibody was only detected when *Trichosporon beigelii* (now classified as *T*. *asahii*) was assessed [13]. Subsequent studies have confirmed the presence of a GXM-like glycan in *T*. *asahii* [14,15] and *T*. *mucoides* [16]. Both species were recognized by a monoclonal antibody to cryptococcal GXM, and the isolated polysaccharides were efficiently incorporated into the cell surface of a *C*. *neoformans* acapsular mutant [15,16]. In both *T*. *asahii* and *T*. *mucoides*, the GXM-like glycan participated in the interaction of the fungi with macrophages, as widely reported for *Cryptococcus* (reviewed in [2]). However, comparative phagocytosis assays revealed that *T*. *mucoides* and *T*. *asahii* were similarly recognized by macrophages, but this process did not depend on TLR2 and CD14 [16], which are some of the typical receptors of cryptococcal GXM [2]. As for the polysaccharide dimensions, *T*. *mucoides* produced smaller molecules than *T*. *asahii* [15,16], and the molecules produced by both species were generally smaller than those produced by cryptococci [10]. *T*. *mucoides* was less virulent in *Galleria mellonella* than *T*. *asahii* [16], raising questions about the relationship between GXM properties and pathogenic mechanisms in *Trichosporon*, which remain to be determined.

**Fig 2:**
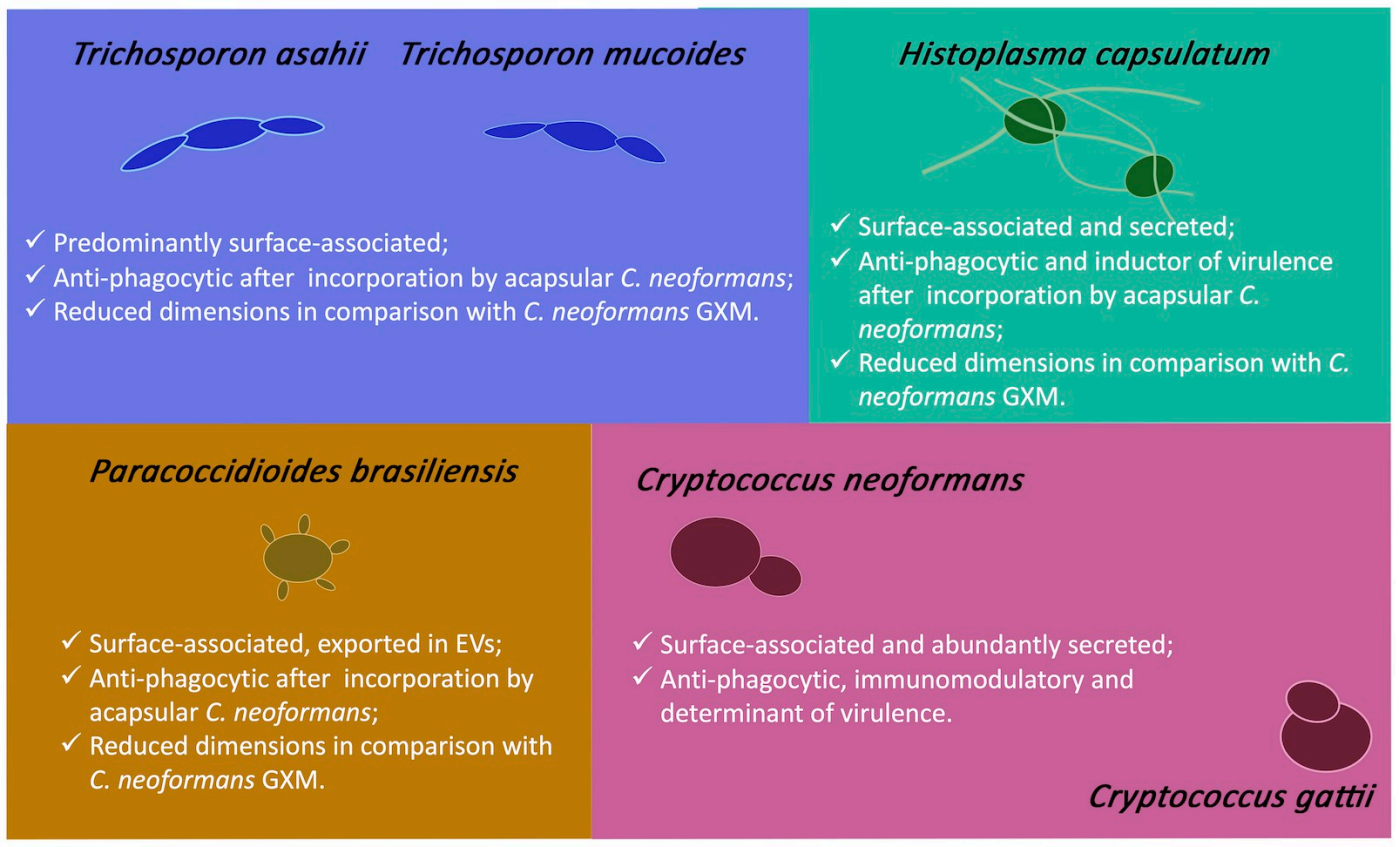
Properties and distribution of GXM-like glycans in fungal pathogens. Glycans with structural and functional similarities to cryptococcal GXM were described in *T*. *asahii*, *T*. *mucoides*, *H*. *capsulatum*, and *P*. *brasiliensis*. The most prominent functions of GXM-like molecules are summarized in each panel.

GXM-like glycans were found in *Histoplasma capsulatum* [17,18]. In this pathogen, α-1,3 glucan, which is required for capsule anchoring in *Cryptococcus* [4], was necessary for interaction with GXM-like glycans [18]. In vivo and in vitro assays showed that *H*. *capsulatum* was able to incorporate extracellular glycans from *C*. *neoformans* in vivo, increasing its virulence potential during *G*. *mellonella* infection [18]. The glycans were antiphagocytic and could be incorporated by *H*. *capsulatum* even within macrophages [18]. The cell surface of *H*. *capsulatum* was also recognized by an antibody to cryptococcal GXM, although differences in antibody reactivity were observed among different strains [17]. Like in the *Trichosporon* genus, extracellular glycans of *H*. *capsulatum* were incorporated by an acapsular mutant of *C*. *neoformans*, resulting in increased survival of the fungus after ingestion by macrophages [17]. In *Paracoccidoides brasiliensis*, surface glycans were recognized by 3 different monoclonal antibodies raised to cryptococcal GXM [19]. When compared to *Cryptococcus*, the size of the glycans produced by *P*. *brasiliensis* had smaller molecular diameters, but they were efficiently used by an acapsular mutant of *C*. *neoformans* as a surface coat [19]. Mutants that incorporated the *P*. *brasiliensis* glycans had reduced interaction with macrophages in vitro [19]. Taken together, these results indicate that the impact of GXM-like molecules can go far beyond what is known in *Cryptococcus*. These findings are summarized in Fig 2.

## 4. Possible venues for future studies

Polysaccharides have been understood as structural components of the fungal surface for decades. However, the characterization of their multiple, sophisticated immunological functions has elevated these molecules as efficient modulators of the immune response to fungal diseases [20]. Nonetheless, the diversity observed for cryptococcal GXM raises questions regarding the most appropriate polysaccharide preparations to stimulate immune responses. As discussed in this essay, GXM can exert variable and even contradictory functions depending on its molecular mass, confirming previous observations with chitin. In this system, large chitin fragments were immunologically inert, while both intermediate-sized chitin (40 to 70 microm) and small chitin (<40 microm, largely 2 to 10 microm) stimulated TNF elaboration [21]. In fact, chitin samples contain size-dependent pathogen-associated molecular patterns that stimulate TLR2, dectin-1, and the mannose receptor and induce the production of pro- and anti-inflammatory cytokines [21], among other effects. Similar variability was observed for other fungal polysaccharides [22], strongly suggesting that specific structural properties require consideration for the selection of immunologically active glycans produced by fungi.

Regarding the structural characterization of GXM and other fungal polysaccharides, it is now clear that aspects other than composition, glycosidic linkages, and branching demand attention. For instance, in this manuscript, we discuss how the molecular mass of fungal glycans can impact their functions. Variabilities in the molecular mass of polysaccharides cannot be detected by the methods conventionally used for determining composition and glycosidic linking between sugar residues, suggesting the need for additional ways of determining the structure of fungal polysaccharides. In summary, we propose that the diversity of cryptococcal GXM and other fungal polysaccharides is much higher than initially thought, and investigating less explored aspects of these molecules can reveal unpredicted functions.
